# Comparison of Propofol and Sevoflurane Anaesthesia in Terms of Postoperative Nausea-Vomiting Complication in Cardiac Surgery Patients Undergoing Enhanced Recovery After Surgery Protocol: A Prospective Randomized Study

**DOI:** 10.4274/TJAR.2024.241622

**Published:** 2024-07-12

**Authors:** Aslıhan Aykut, Nevriye Salman, Zeliha Aslı Demir, Ayşegül Özgök, Serdar Günaydın

**Affiliations:** 1 University of Health Sciences Turkey, Ankara Bilkent City Hospital, Clinic of Anaesthesiology and Reanimation, Ankara, Turkey; 2 University of Health Sciences Turkey, Ankara Bilkent City Hospital, Clinic of Cardiovascular Surgery, Ankara, Turkey

**Keywords:** Cardiac surgery, ERAS, postoperative nausea-vomiting, propofol, sevoflurane

## Abstract

**Objective:**

Postoperative nausea (PN) and vomiting (PONV) in cardiac surgery increases adrenergic stimulation, limits mobilization and oral intake, and can be distressing for patients. The primary aim of our study was to investigate the effect of sevoflurane and propofol anaesthesia on the incidence of PONV in cardiac surgery patients undergoing Enhanced Recovery After Surgery (ERAS) protocol.

**Methods:**

Following ethics committee approval, 62 patients undergoing elective coronary artery bypass surgery with ERAS protocol were included in this prospective randomized study. After standard induction of anaesthesia, Group S received 1.5-2% sevoflurane and Group P received 50-100 µg kg^-1^ min^-1^ propofol infusion as maintenance anaesthetic agent with a bispectral index of 40-50. The incidence of PN and PONV between 0-6 hours (early) and 6-24 hours (late) after extubation was compared as the primary outcome. The incidence of delirium was analyzed as a secondary outcome for similar periods.

**Results:**

In the propofol group, 3 patients were excluded due to postoperative tamponade revision and prolonged mechanical ventilation. PN in the early post-extubation period (29% vs. 7.1%, *P*=0.031) was significantly higher in Group S. The incidence of delirium was similar between the groups in both periods.

**Conclusion:**

Propofol may reduce the incidence of PN in the first 6 hours after extubation compared with sevoflurane. We believe that this period will be beneficial for gastrointestinal tolerance as it is the period when oral intake is initiated in patients. In conclusion, propofol maintenance in cardiac surgery patients may facilitate patient rehabilitation as part of the ERAS protocol.

**Main Points**

⦁ The Enhanced Recovery After Surgery (ERAS) protocol is a set of evidence-based practices that accelerate the healing process of patients. One of these is the initiation of oral intake and transition to physiological nutrition as soon as possible. One of the obstacles to this is postoperative nausea and vomiting (PONV).

⦁ Propofol-based anaesthesia maintenance may be preferred in patients at risk of PONV undergoing cardiac surgery. When PONV decrease, patient rehabilitation becomes easier and this may contribute as a gain within the scope of the ERAS protocol.

## Introduction

Postoperative nausea (PN) and vomiting (PONV) is frequently encountered following general anaesthesia, causing discomfort and complaints in patients.^1^ Sometimes PONV can lead to significant postoperative complications such as aspiration pneumonia, increased intracranial pressure.^1, 2^ It is known that the frequency of PONV is around 80% in high-risk populations and 30% in the general population. Increased medical costs, prolonged hospital stays, and hospital readmissions are common in cases of PONV.^1^ Several risk factors have been associated with PONV, including female gender, non-smoking status, previous history of PONV, motion sickness, young age, certain types of surgery, prolonged anaesthesia, use of nitrous oxide, postoperative opioids and volatile anaesthetics.^2, 3^

Enhanced Recovery After Surgery (ERAS) is an initiative that aims to develop multimodal, interdisciplinary care to support the perioperative recovery of patients undergoing surgery.^4^ This strategy focuses on reducing complications and allowing patients to return to normal activities sooner. ERAS protocols place great emphasis on PONV, which affects 20% to 67% of patients in cardiac surgery, increases adrenergic stimulation, limits mobilization and oral intake, and can be distressing for patients.^5^ Within the scope of the ERAS protocol in cardiac surgery, methods such as routine antiemetic treatment, short fasting periods and early nutrition are applied to prevent and/or treat the development of PONV.^6, 7^

Another important part of the ERAS protocol is early routine delirium screening in the postoperative period.^6^ Studies in non-cardiac surgeries have found very different results on the effects of inhalation and propofol anaesthesia maintenance on the incidence of postoperative delirium.^8, 9^ The pathogenesis of delirium in cardiac surgery is more complicated with the addition of many factors such as cardiopulmonary bypass (CPB), hypothermia-rewarming, preoperative anxiety, prolonged mechanical ventilation, comorbidities. Optimizing these factors with the Enhanced Recovery After Cardiac Surgery (ERACS) program can exclude confounding factors in the relationship between anaesthesia maintenance and delirium.

To our knowledge, the relationship between PONV and maintenance of anaesthesia in patients undergoing ERAS protocol in cardiac surgery has not been investigated before. It is known that propofol has a direct antiemetic effect, but on the contrary, the risk of nausea and vomiting increases with inhalation anaesthesia.^10^ The hypothesis of our study is that the residual effects of anaesthetic agents are evident in the first 24 hours postoperatively, and therefore, anaesthesia with propofol causes less PONV compared to sevoflurane.

The primary aim of our study is to compare the effects of propofol and sevoflurane anaesthesia on PONV complications in patients undergoing coronary surgery who underwent ERAS protocol. In addition, the secondary aim is to determine the relationship between these two different anaesthesia maintenance and postoperative delirium.

## Methods

**Figure 1:**
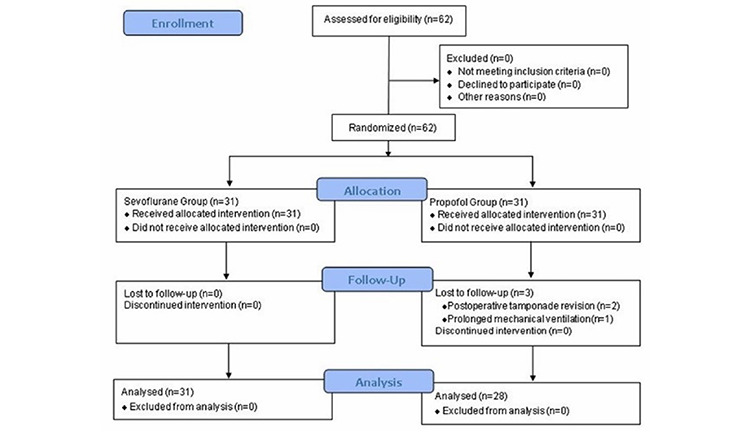
Flow diagram

This prospective randomized study was conducted according to Consolidated Standards of Reporting Trials guidelines^11^ and approved by the University of Health Sciences Tutkey, Ankara Bilkent City Hospital No. 1 Clinical Research Ethics Committee (approval no.: E1-23-3601, date: 07.06.2023). Study included 62 adult male patients with American Society of Anesthesiologists (ASA) physical status II or III, scheduled for elective on-pump coronary artery bypass grafting (CABG) with median sternotomy under the ERAS program (Figure 1). Emergency surgery, redo or off-pump surgery, intolerance to one of the study drugs, female gender, ejection fraction <%40, obese patients (body mass index >30 kg m^-2^), history of motion sickness, history of antiemetic use including steroids in the last two weeks, gastro-esophageal reflux disease, neurological disease (e.g., Parkinson’s, mental retardation), psychiatric disease (e.g., major depressive disorder, schizophrenia,), failed fascial plan block before induction and lack of informed consent were determined as exclusion criteria. Patients were evaluated at the anaesthesia visit the night before surgery and written informed consent was obtained. In the preoperative period, all patients received peroral pregabalin 150 mg (12 hours before) and intravenous cefazolin sodium 1000 mg (30 minute before) according to the ERAS protocol. Twenty-four hours before surgery, the patients were visited by a physiotherapist and respiratory exercises were implemented. Patients were fasted for 6 hours for solid foods and 2 hours for clear liquids. Also were given 400 mL 12.5% maltodextrin 2 hours before surgery. Maltodextrin solution was also given to diabetic patients, but a different insulin protocol was applied to them with the recommendation of endocrine clinicians.

Before anaesthesia induction, bilateral erector spinae plane (ESP) block (ESP, 40 mL 0.25% bupivacaine, 20 mL) was applied to the patients in the prone position under ultrasound guidance (PHILIPS Affiniti 50 color Doppler ultrasound device, Philips L12-5 50 mm linear array transducer). Local anaesthetic was applied to the lateral T5-6 spinous processes with a single shot method without inserting a catheter, a total of 40 mL on both sides. Afterwards, patients were turned to supine position and pulse oximetry, five-channel electrocardiography, invasive arterial blood pressure and bispectral index monitoring (BIS™, Covidien, MN, USA) were performed. Sensory blockade of ESP block was controlled; failed block was not observed in any patient. Anaesthesia induction was performed with intravenous propofol (2-2.5 mg kg^-1^), fentanyl (1 µg kg^-1^), rocuronium (0.8 mg kg^-1^) and lidocaine (1 mg kg^-1^).  After endotracheal intubation, ventilation was adjusted to end tidal carbon dioxide 35-40 mmHg and an internal jugular venous catheter was inserted under ultrasound guidance. For the selection of the agent to be used for maintenance of anaesthesia, the patients were divided into groups using a computer-generated random number sequence. Anaesthesia was maintained by titrating 1.5-2% sevoflurane in Group S and 50-100 µg kg^-1 ^min^-1^ propofol infusion in Group P to achieve a bispectral index between 40-50. Sevoflurane was continued to be administered during the CPB period by means of a vaporizer integrated into the heart-lung machine. Both groups received a remifentanil infusion of 0.05-to-0.25 µg kg^-1 ^min^-1^ throughout the operation, the dose was increased during CPB, rocuronium added when necessary.^12^

After sufficient activated clotting time (>480 s), aortic and venous cannulations were performed and CPB was started with retrograde autologous priming. CPB was performed at moderate hypothermia (28-31 °C). The target pump flow rate was 2-2.5 L min m^-2^. During the operation, erythrocyte suspension was administered if the hemoglobin concentration was below 7.5 g dL^-1^ and insulin infusion was administered if glucose was above 200 mg dL^-1^. Lidocaine 1 mg kg^-1^ and magnesium 1500 mg were administered during aortic clamp removal, and heparin was reversed with protamine in a 1:1 ratio at the end of CPB. Paracetamol (1 g), fentanyl 1 µg kg^-1^ and ondansetron 4 mg were given to both groups of patients during sternal closure. Patients were sedated with dexmedetomidine (0.2-0.7 µg kg^-1 ^h^-1^) until extubation in the intensive care unit (ICU). Postoperative analgesia was maintained with intravenous paracetamol (1 g) every 8 hours in both groups. In cases where postoperative analgesia was not sufficient (visual analogue scale >4), tramadol (0.5-1 mg kg^-1^) was given as a rescue analgesic in both groups.

The primary and secondary outcomes of the study were evaluated in the first 0 to 6 hours (early) after extubation, when the residual effects of anaesthetics are intense, and in the 6 to 24 hours (late) periods, when they are less intense. The surgical team assessing postoperative complications was blinded to the method of intraoperative anaesthesia. Nausea or retching alone was defined as PN. Nausea accompanied by vomiting was defined as PONV. In case of PONV, ondansetron 4 mg (at least 6 hours after the previous dose) was given as a rescue antiemetic. The incidence of PN and PONV was determined by the number of patients who experienced nausea and/or vomiting during the 24-hour. Within the scope of ERAS, oral intake began as soon as the swallowing reflex was restored (often immediately after extubation). Patients were evaluated with the Nursing Delirium Screening Scale (Nu-DESC) in the early and late periods, and patients with a Nu-DESC score ≥2 were also considered delirium.^13^ Extubation, ICU and hospitalization times were also recorded.

The minimum required sample size with 95% power at a significance level of 5% was obtained with 28 patients in each group, with reference to a study reporting a PN rate of 13.3% in patients receiving propofol, compared with 53.3% for sevoflurane.^14^ Considering the possibility of exclusion, 31 patients per group were included in the study. Since anaesthetics and PONV in ERACS patients is a subject that has not been investigated before, existing studies were used as reference despite limitations. Therefore, we believe that the incidence values of this study will be a reliable reference for subsequent studies.

### Statistical Analysis

The IBM SPSS.26.0 software was used for all dates analyzed. Descriptive statistics were presented as absolute numbers (n) and percentages (%) for categorical variables, the median-interquartile range (25^th^-75^th^ percentiles) for non-normally distributed data, and the mean ± standard deviation for normally distributed data. Categorical variables were compared using χ^2^ or Fisher’s exact test. Continuous variables between two groups were compared using Mann-Whitney U or independent samples t-test, based on a Kolmogorov-Smirnov test for normality. For the overall incident rate, a Fisher’s exact test was used to estimate the relative risk and 95% confidence interval (CI) of incidence. For all analyses, *P *< 0.05 was considered statistically significant.

## Results

From May 2023 to June 2023, a total of 62 adult patients who underwent elective CABG with CPB under the ERAS protocol in our cardiac center were included the study, statistical analysis was completed with 59 patients. In the propofol group, 2 patients were excluded from the study due to surgical bleeding requiring surgery in the postoperative period and 1 patient due to the need for mechanical ventilation for more than 12 hours. There was no significant difference between the two groups in terms of demographic data, ASA physical status, comorbidities and preoperative laboratory data (Table 1).

**Figure 2:**
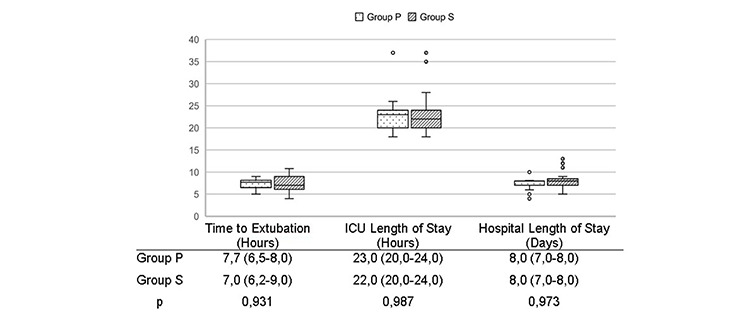
Extubation time, length of ICU and hospital stay of the groups. The Mann-Whitney U test was used for continuous variables (median, IQR); box plot represent data as median values (bold horizontal line) and interquartile range (box) ICU, intensive care unit; IQR, interquartile range

The total intraoperative remifentanil requirement was significantly lower in the sevoflurane group (*P*=0.001). The duration of cross clamp, CPB and operation, total intravenous fluid volume and urine output, blood and blood product transfusion, inotropic and vasopressor medication requirements were similar between the groups (Table 2). There was no difference in terms of extubation time (*P*=0.931), ICU (*P*=0.987), and hospital (*P*=0.973) length of stay (Figure 2). There was no 30-day mortality in the study patients.

Within the first six hours after extubation, PN occurred in 18.6% and PONV in 10.1% of all patients. In the same time interval, the incidence of nausea was significantly lower in the propofol group compared to sevoflurane group (7.1% and 29%, respectively, *P*=0.031). The relative risk and 95% CI for propofol anaesthesia was found as 0.24 (0.05-1.04) for PN (*P*=0.031). In the 6-24 h late period, the incidence of PN was similar between the groups and never encountered vomited (Table 3). As secondary outcome in our study, there was no difference between the groups in terms of delirium scores assessed by Nu-DESC in the early and late periods.

## Discussion

In this study, lesser nausea was observed in the first 6 h after extubation with the use of propofol in anaesthesia maintenance in patients undergoing CABG with the ERAS protocol, no difference was found in terms of PONV. In the late period (6-24 h), it was not found difference between the groups in parameters PN and PONV. Besides, with regard to delirium, no difference was found between the groups in the early and late periods.

Anaesthesia maintenance in cardiac surgery has been the subject of numerous studies. Organs protection seems to be the most important factor in the choice of anaesthetic management. Volatile anaesthetics protect the myocardium from ischemic damage by decreasing myocardial oxygen demand and increasing oxygen supply through moderate vasodilation.^15^ The assumption of improvement in mortality and morbidity due to myocardial protection through preconditioning causes volatile agents to be preferred in cardiac surgery.^16^ Similarly, preclinical studies have shown that propofol attenuates myocardial ischemia-reperfusion injury (IRI) by inhibition of the Wnt/β-catenin signaling pathway and blocking autophagy.^17^^, ^^18^ However, myocardial ischemia is inevitable when aortic cross-clamping is performed in on-pump cardiac surgery and its severity depends more on the severity of the underlying disease, duration of cross-clamping and myocardial protection with cardioplegia.^19^Clinical studies have shown that the use of volatile anaesthetics in cardiac surgery has no superiority over propofol in myocardial infarction, hospital readmission, short-term or one-year mortality.^20^^, ^^21^ Ştefan et al.^20^ suggest that IRI and mortality in cardiac surgery are too complex to be reduced solely to the choice of anaesthesia regimen. It is more valuable to reveal the relationship between minor outcomes such as nausea and vomiting and maintenance of anaesthesia, rather than discovering highly dependent outcomes such as mortality.

PN is described by patients as the most distressing complication of anaesthesia.^22^ The occurrence of PONV is thought to be a multifactorial complication involving operative, anaesthetic, and patient-specific risk factors.^23^ In this study, in order to examine the isolated effect of anaesthesia management on PN and PONV, it was adjusted the study group from CABG surgery male patients who underwent ERAS protocol which minimized other risk factors. In scope of this protocol, ESP block and opioid- reducing analgesia method such as paracetamol were applied in all patients. In addition, in the preoperative period, patients were given carbohydrates two hours before surgery to eliminate the effect of catabolism and empty stomach that would cause nausea and vomiting. Routine antiemetics were administered while closing the sternum at the end of the operation. In the light of these nausea and vomiting preventive measures applied to both groups, nausea was seen more frequently in the early period in patients who were maintained with sevoflurane (7.1% vs. 29%).

In a systematic review and meta-analysis, the incidence of PONV following outpatient surgery was found lower in patients receiving propofol than in patients receiving volatils.^24^ In a study in which patients at high risk for postoperative PONV were included, the 72-hour cumulative PONV was found 46% in total intravenous anaesthesia group with propofol, 60% in volatile group (isoflurane + nitrous oxide), and the highest PONV was observed in the post-anaesthesia care unit period.^25^ Undoubtedly, the incidence of PONV was found high in that study due to reasons such as the inclusion of high-risk patients, the use of nitrous oxide, and the three-day cumulative incidence. The low incidence of PONV in our study is related to the application of the ERAS protocol, the inclusion of only male patients, the fact that patients were intubated in the first 6 hours when postoperative PONV was highest, and the use of opioid-sparing multimodal analgesia. A Randomized controlled study examining risk factors for PONV, effects of volatile anaesthetics was found to be dose-dependent and also several times riskier than all other PONV risk factors (including lack of routine antiemetic use) in the early postoperative period.^26^ In line with this in our study, the incidence of nausea was found higher in the sevoflurane group in the first 6 hours. This may be explained by the pharmacokinetic profile of propofol, where therapeutic anti-emetic plasma levels are unlikely to persist in the late period after anaesthesia administration because of its short half-life.^24^ However, although the low incidence of PONV associated with propofol has been attributed to the antiemetic property of propofol, no relationship has been found between PONV and the degree of exposure to propofol, but in contrast, volatile anaesthetics have a pro-emetic effect in proportion to the degree of exposure.^26^ Therefore, the question arises whether the difference between sevoflurane and propofol anaesthesia is due to the antiemetic properties of propofol or the emetogenic properties of volatile anaesthetics? In any case, it may be more logical to avoid inhalation anaesthesia instead of adding an antiemetic to prevent PN in high-risk patients.

In cardiac surgery, preoperative oral nutrition support with carbohydrate-based beverages as well as early postoperative feeding is associated with shorter hospitalization and ICU length of stay.^27^ Delayed initiation of nutritional support in surgical intensive care patients leads to delayed restoration of gastrointestinal activity and energy deficits.^28^ Therefore, early postoperative enteral nutrition is an essential component of the ERAS protocol and, it is recommended to start early oral nutrition after the return of the swallowing reflex is confirmed.^29^^, ^^30^ Decreasing effect of propofol anaesthesia on the incidence of early PN may facilitate patients to start oral nutrition in this period. This may lead to earlier recovery and shorter ICU stay.

As part of the ERAS program, routine postoperative delirium screening is recommended to diagnosed and early treatment.^6^ Delirium after cardiac surgery is associated with decreased in-hospital and long-term survival, increased hospital readmission, and poor cognitive and functional recovery.^31^ Determining the risk factors of delirium is also important to identify preventable causes. According to various preclinical and animal studies, it has been reported that inhalation anaesthetics cause neurodegeneration, whereas propofol causes less cognitive impairment with a strong anti-inflammatory effect.^32, 33, 34, 35^ However, in numerous clinical studies postoperative delirium after cardiac surgery has not been found different between patients receiving sevoflurane and propofol anaesthesia.^36, 37, 38^ Risk factors for delirium after cardiac surgery include advanced age, dementia, prolonged CPB duration, high perioperative transfusion requirement, low preoperative albumin level, high postoperative C-reactive protein concentration and longer ICU stay.^39^^, ^^40^ In our study, there were no confounding factors such as advanced age, emergency surgery and low preoperative albumin and, similar to the literature, postoperative delirium did not differ in patients receiving propofol versus sevoflurane. It was also considered that interventions such as pregabalin administration, multimodal analgesia and early extubation within the scope of the ERAS protocol also can contribute to the low incidence of delirium.

### Study Limitations

Since this study was conducted using data from a single center, its generalizability is limited. In our study, PONV was evaluated quantitatively and not graded (present/absent). Although Nu-DESC is assessed by well-trained ICU nurses, the hypoactive form of delirium may have been overlooked because the fully hypoactive form of delirium is generally more difficult to detect than the hyperactive form. Additionally, the study referred for sample analysis included patients with a higher risk of PONV (our patient group was at lower risk). Therefore, studies planned with a larger number of patient groups are needed.

## Conclusion

In conclusion, it is expected that small gains in cardiac ERAS applications will accumulate and turn into large gains, therefore, reducing PONV, which is a significant discomfort, by choosing a patient-specific anaesthetic is a valuable result. Propofol-based anaesthesia maintenance may be preferred in patients at risk for PONV who will undergo cardiac surgery. When PONV decreases, patient rehabilitation becomes easier and this may contribute as a gain within the scope of the ERAS protocol.

## Ethics

**Ethics Committee Approval:** This study was approved by the University of Health Sciences Turkey, Ankara Bilkent City Hospital No. 1 Clinical Research Ethics Committee (approval no.: E1-23-3601, date: 07.06.2023).

**Informed Consent:** Patients were evaluated at the anaesthesia visit the night before surgery and written informed consent was obtained.

## Figures and Tables

**Table 1. Demographic Data, Comorbidities and Preoperative Laboratory Data:** 

	**Group P (n = 28)**	**Group S (n = 31)**	*P* value*
Age (years), Mean ± SD	60.50±5.7	61.64±9.3	0.571
BMI (kg m^2-1^), Mean ± SD	29.14±3.5	28.47±4.5	0.533
ASA physical status (II/III), n (%)	8/20 (28.6/71.4)	12/19 (38.7/61.3)	0.411
Smoker, n (%)	16 (57.1)	11 (35.5)	0.095
Diabetes mellitus, n (%)	12 (42.9)	15 (48.4)	0.670
Hypertension, n (%)	20 (71.4)	20 (64.5)	0.570
COPD, n (%)	4 (14.3)	2 (6.5)	0.320
LVEF (%), Mean ± SD	53.50±8.7	52.93±7.4	0.792
**Preoperative laboratory data**			
Hemoglobin (gr dL^-1^), Mean ± SD	13.85±1.8	13.46±2.1	0.447
White blood cell (10^3^ uL), Mean ± SD	8.01±2.5	8.88±2.5	0.197
Platelet, (10^3^ uL), Mean ± SD	254.00±94.0	248.77±56.5	0.800
HbA1c, (%), Mean ± SD	7.30±2.1	7.18±1.8	0.820
Creatinine, (mg dL^-1^), Mean ± SD	0.94±0.2	0.99±0.2	0.368

^*^The independent samples t-test was used for continuous variables; the χ^2^ was performed for categorical variables.

ASA, American Society of Anesthesiologists; BMI, body mass index; COPD, chronic pulmonary disease; LVEF, left ventricular ejection fraction; SD, standard deviation.

**Table 2. Intraoperative Data:** 

	**Group P (n = 28)**	**Group S (n = 31)**	*P* value*
Remifentanil (mg), Mean ± SD	4.12±1.7	2.84±1.0	0.001
Propofol (mg), Mean ± SD	1128.57±363.9	-	-
Sevoflurane (mL), Mean ± SD	-	57.96±31.87	-
CC time (min), Mean ± SD	79.46±27.4	68.16±19.5	0.072
CPB time (min), Mean ± SD	118.07±34.8	104.09±22.4	0.070
Operation time (min), Mean ± SD	322.85±71.6	306.93±60.8	0.360
Crystalloid (mL), Median (IQR)	1625.00 (1500.0-1900.0)	1700.00 (1300.0-2000.0)	0.537
Urine output (mL), Median (IQR)	850.00 (600.0-1200.0)	800.00 (600.0-1100.0)	0.964
**Red blood cell transfusion, n (%)**			0.799
None	20 (71.4)	23 (76.7)	
1 Unit	2 (7.1)	3 (10.0)	
2 Units	2 (7.1)	2 (6.7)	
3 Units	4 (14.3)	2 (6.7)	
**Fresh frozen plasma use, n (%)**			0.135
None	26 (92.9)	28 (93.3)	
1 Unit	0 (0.0)	2 (6.7)	
3 Units	2 (7.1)	0 (0.0)	
Platelet concentrates, 1 unit, n (%)	2 (7.1)	0 (0.0)	0.136
Dopamine, n (%)	8 (28.6)	10 (32.3)	0.759
Dobutamine, n (%)	2 (7.1)	3 (9.7)	0.727
Norepinephrine, n (%)	6 (21.4)	6 (19.4)	0.843

^*^The independent samples t-test and Mann-Whitney U test were used for continuous variables; the χ^2^ was performed for categorical variables.

CC, cross clamp; CPB, cardiopulmonary bypass.

**Table 3. Postoperative Outcomes:** 

	**Group P (n=28)**	**Group S (n=31)**	Relative risk^*^ (95% CI)	*P* value^**^
**Post-extubation 0-6. hours**				
Pain VAS >4, n (%)	10 (35.7)	12 (38.7)	0.94 (0.57-1.54)	0.513
Delirium (Nu-DESC ≥2), n (%)	0 (0)	2 (6.5)	-	0.272
Incidence of nausea, n (%)	2 (7.1)	9 (29.0)	0.24 (0.05-1.04)	**0.031**
Incidence of PONV, n (%)	2 (7.1)	4 (12.9)	0.55 (0.11-2.79)	0.386
**Post-extubation 6-24. hours**				
Pain VAS >4, n (%)	4 (14.3)	8 (25.8)	0.55 (0.18-1.64)	0.272
Delirium (Nu-DESC ≥2), n (%)	2 (7.1)	3 (9.7)	0.73 (0.13-4.10)	0.549
Incidence of nausea, n (%)	2 (7.1)	6 (19.4)	0.36 (0.08-1.68)	0.162
Incidence of PONV, n (%)	0 (0.0)	0 (0.0)	-	-
Patients requiring rescue analgesics in the first 24 hours, n (%)	10 (35.7)	15 (48.4)	(0.74-2.35)	0.325

^*^Relative risk in the incidence of PONV for propofol versus sevoflurane.

^**^χ^2^ test or Fisher’s exact test.

PONV, postoperative nausea and vomiting; Nu-DESC, nursing delirium screening scale; VAS, visual analogue scale; CI, confidence interval.
